# SIMPA: an open-source toolkit for simulation and image processing for photonics and acoustics

**DOI:** 10.1117/1.JBO.27.8.083010

**Published:** 2022-04-04

**Authors:** Janek Gröhl, Kris K. Dreher, Melanie Schellenberg, Tom Rix, Niklas Holzwarth, Patricia Vieten, Leonardo Ayala, Sarah E. Bohndiek, Alexander Seitel, Lena Maier-Hein

**Affiliations:** a German Cancer Research Center (DKFZ), Division of Intelligent Medical Systems, Heidelberg, Germany; b Heidelberg University, Faculty of Physics and Astronomy, Heidelberg, Germany; c Heidelberg University, Faculty of Mathematics and Computer Science, Heidelberg, Germany; d HIDSS4Health - Helmholtz Information and Data Science School for Health, Heidelberg, Germany; e Heidelberg University, Medical Faculty, Heidelberg, Germany; f University of Cambridge, Cancer Research UK Cambridge Institute, Robinson Way, Cambridge, United Kingdom; g University of Cambridge, Department of Physics, Cambridge, United Kingdom

**Keywords:** simulation, open-source, photoacoustics, optical imaging, acoustic imaging

## Abstract

**Significance:**

Optical and acoustic imaging techniques enable noninvasive visualisation of structural and functional properties of tissue. The quantification of measurements, however, remains challenging due to the inverse problems that must be solved. Emerging data-driven approaches are promising, but they rely heavily on the presence of high-quality simulations across a range of wavelengths due to the lack of ground truth knowledge of tissue acoustical and optical properties in realistic settings.

**Aim:**

To facilitate this process, we present the open-source simulation and image processing for photonics and acoustics (SIMPA) Python toolkit. SIMPA is being developed according to modern software design standards.

**Approach:**

SIMPA enables the use of computational forward models, data processing algorithms, and digital device twins to simulate realistic images within a single pipeline. SIMPA’s module implementations can be seamlessly exchanged as SIMPA abstracts from the concrete implementation of each forward model and builds the simulation pipeline in a modular fashion. Furthermore, SIMPA provides comprehensive libraries of biological structures, such as vessels, as well as optical and acoustic properties and other functionalities for the generation of realistic tissue models.

**Results:**

To showcase the capabilities of SIMPA, we show examples in the context of photoacoustic imaging: the diversity of creatable tissue models, the customisability of a simulation pipeline, and the degree of realism of the simulations.

**Conclusions:**

SIMPA is an open-source toolkit that can be used to simulate optical and acoustic imaging modalities. The code is available at: https://github.com/IMSY-DKFZ/simpa, and all of the examples and experiments in this paper can be reproduced using the code available at: https://github.com/IMSY-DKFZ/simpa_paper_experiments.

## 1 Introduction

Optical and acoustic imaging techniques enable real-time and noninvasive visualisation of structural and functional tissue properties without exposing the patient to harmful ionizing radiation. Nevertheless, the applicability of purely optical and acoustic imaging techniques is limited, for example, by the low penetration depth of near-infrared spectroscopy^1^ or by the difficulties of measuring functional tissue properties with ultrasound imaging.^2^ Furthermore, quantitative measurements are challenging as the state-of-the-art model-based approaches to solve the underlying inverse problems rely on assumptions that might not hold when applied to *in vivo* measurements.

Data-driven approaches can be chosen to address these inverse problems. To this end, high-quality well-annotated data are needed, for example, to train deep learning algorithms^3^^–^^5^ or to optimize device design.^6^^,^^7^ In living subjects, the acquisition of such data is extremely difficult because the underlying optical and acoustic tissue properties are generally not well known.^8^ As such, for algorithm training, many researchers instead use simulated data, which are comparatively easy to obtain, have known underlying optical and acoustic properties, and can be used for both algorithm training and validation.^9^^–^^13^ Nevertheless, the application of algorithms trained exclusively on synthetic training data to experimental measurements is challenging due to systematic differences between synthetic and experimental data.^14^

Photoacoustic imaging (PAI) combines the advantages of optical and acoustic imaging by exploiting the photoacoustic (PA) effect, resulting in optical contrast with scalable high spatial resolution down to microns as a function of imaging depth, which can be up to several centimeters.^15^ PAI enables the recovery of functional tissue properties, such as blood oxygen saturation.^16^ To quantitatively recover such parameters, two inverse problems have to be solved: the acoustic inverse problem, which constitutes the accurate and quantitative reconstruction of the initial pressure distribution, and the optical inverse problem, which constitutes the quantitative recovery of the optical absorption coefficient.^8^ To generate realistic PA simulations for the purpose of training a data-driven method, all physical and computational aspects of signal formation need to be considered;^17^ these include synthetic volume generation, photon propagation, acoustic wave propagation, and image reconstruction.

In recent years, a heterogeneous software landscape has emerged with various open-source or free-to-use tools to cover each of these physical and computational aspects. For example, for volume generation, there exist open access resources, such as the Digimouse^18^ annotated digital mouse phantom, digital breast phantoms (available at: https://github.com/DIDSR/VICTRE, last visited March 22, 2022),^19^^,^^20^ and the multimodal imaging-based detailed anatomical model of the human head and neck atlas MIDA.^21^ But usually, researchers use pseudorandom distributions of light-absorbing molecules (chromophores) to create tissue-mimicking *in silico* phantoms.^13^^,^^22^ For optical modeling of photon transport in tissue, numerous approaches have been established; these focus in general either on (1) Monte Carlo methods including, for example, mcxyz,^23^ MCX,^24^ or ValoMC,^25^ which uses a Monte Carlo approach to light transport to simulate the propagation of photons in heterogeneous tissue, or (2) analytical methods to solve the radiative transfer equation, including diffusion approximation or finite element solvers as implemented in, for example, NIRFAST^26^ or Toast++.^27^ For acoustic modeling, there exists the popular k-Wave^28^ toolbox, which is a third-party MATLAB toolbox for the simulation and reconstruction of PA wave fields and is one of the most frequently used frameworks in the field. For image reconstruction, there are many different approaches, including backprojection algorithms,^29^^–^^31^ model-based algorithms,^32^^,^^33^ and fast Fourier transform-based reconstruction algorithms.^34^^,^^35^

To navigate these tools and integrate them into a complete pipeline, the user must transform the output of each toolkit into an appropriate form for input to the next^36^^,^^37^ or model the entire process in a joint computational framework.^38^^,^^39^ Each step in assembling these pipelines can be time-consuming or error-prone, especially including correct consideration of the physical quantities and their units. They are typically limited to the toolkits that are currently integrated in their respective framework and thus lack broad applicability to other simulators. Furthermore, a seamless exchange from, e.g., a finite element method optical forward simulator to a Monte Carlo simulator is not straightforward in existing frameworks.

To tackle these challenges, we developed the open-source simulation and image processing for photonics and acoustics (SIMPA) Python toolkit, which features a modular design that allows for easy exchange and combination of simulation pipeline elements. In its first version, the toolkit facilitates the simulation and processing of PA data and provides a straightforward way to adapt a simulation to meet the specific needs of a given researcher or project. It can easily be extended to support simulations corresponding to other optical and acoustic imaging modalities. The core idea of the framework is to standardize the information flow between different computational models by providing a central software architecture that abstracts from the individual requirements of external libraries. SIMPA achieves this by defining abstract implementations of the simulation steps based on adapters that can be implemented, such that specific toolkits can easily be integrated into the SIMPA ecosystem. SIMPA is tested using both Windows (specifically Windows 10) and Linux (specifically Ubuntu 20.04) operating systems. Third-party toolkits are executed on the GPU by default if this is supported by the respective toolkit and a compatible GPU is installed. Furthermore, SIMPA offers the possibility of exporting simulated time-series data compliant to the data format proposed by the International Photoacoustic Standardisation Consortium (IPASC).^40^

In this paper, we first outline the purpose and the software details of SIMPA in Sec. 2. Here, we give an overview of the software development process, the software architecture, the modeling of digital device twins, and computational tissue generation. Afterward, there is an extensive simulation and image processing examples section (Sec. 3) in which we show the possibilities that SIMPA offers. We demonstrate the modularity of SIMPA by showcasing the results of example simulations including an overview of how parameter choices can affect the results and the degree of realism of the simulations that is achievable with SIMPA.

## 2 SIMPA Toolkit

**Fig. 1:**
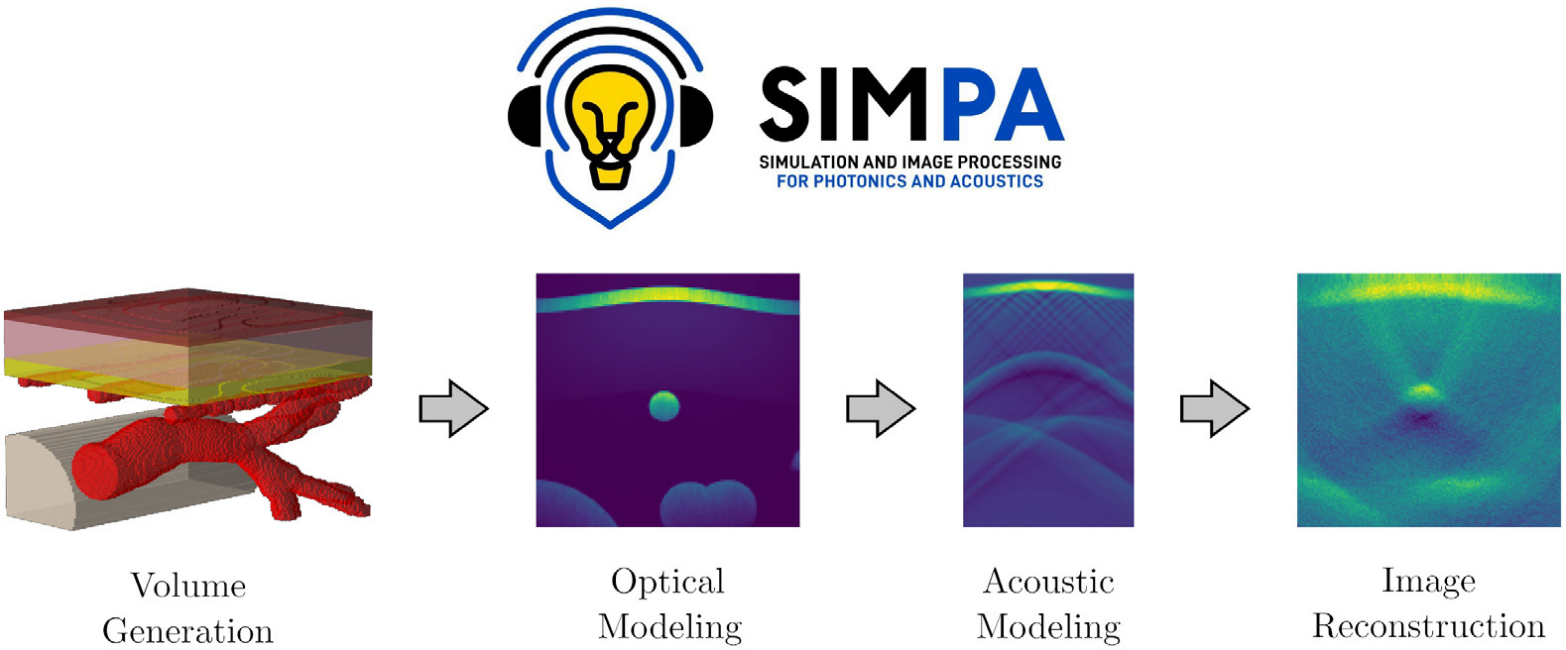
The simulation and image processing for photonics and acoustics (SIMPA) toolkit.

SIMPA aims to facilitate realistic image simulation for optical and acoustic imaging modalities by providing adapters to crucial modeling steps, such as volume generation, optical modeling, acoustic modeling, and image reconstruction (Fig. 1). SIMPA provides a communication layer between various modules that implement optical and acoustic forward and inverse models.

Non-experts can use the toolkit to create sensible simulations from default parameters in an end-to-end fashion. Domain experts are provided with the functionality to set up a highly customisable pipeline according to their specific use cases and tool requirements.

The following high-level requirements are key to meeting the above purpose:

1. Modularity: The different modules of the simulation pipeline should be implemented such that each of them can be paired with arbitrary implementations of preceding or succeeding modules. Specific module implementations can seamlessly be exchanged without breaking the simulation pipeline. The user has the freedom to arrange the elements of the simulation pipeline in exactly the way that they choose.
2. Extensibility: The user should have the freedom to add any custom elements to the pipeline and to implement custom module adapters. There should exist documentation that shows how custom adapters for each module and completely new modules can be implemented.
3. Physical correctness: Each module implementation should have a single purpose, produce plausible results, and not alter other parts of the pipeline. Physical quantities (i.e., units) should be correctly handled by the information flow between separate modules.
4. Independence: Arbitrarily many sequentially executed SIMPA simulations should not influence the results of subsequent simulations.
5. Usability: The entry for new users must be as easy as possible such that sensible PA images can be simulated without prior knowledge. A simulation with default parameters can be started using only a few lines of code.

The following sections of this paper introduce the software development life cycle in Sec. 2.1 and SIMPA’s software architecture in Sec. 2.2, as well as another prominent contribution of SIMPA: a volume creation adapter that enables the user to create diverse spatial distributions of tissue properties as detailed in Sec. 2.4.

### 2.1 Software Development Life Cycle

SIMPA is developed using the Python programming language (Python Software Foundation),^41^ version 3.8 because it is currently one of the most commonly used programming languages. We use git^42^ as the version control system, and the code is maintained on GitHub (available at: https://github.com/IMSY-DKFZ/simpa, last visited March 22, 2022). Stable versions of the develop branch are integrated into the main branch and then form a release with an increase in the version number according to the Semantic Versioning Specification (SemVer) scheme.^43^

SIMPA code is written using a quality-controlled development process. Every feature request or bug fix is assigned an issue on the SIMPA GitHub page (available at: https://github.com/IMSY-DKFZ/simpa/issues, last visited March 22, 2022). Issues can be opened and commented on by any SIMPA user, and the code is written in separate branches that are only integrated into the develop branch after a successful code review by a member of the SIMPA core developer team. To ensure good code quality, the code reviews are designed to check whether the code follows the SIMPA developer guide:

1. The code is executable and yields the expected result in a typical use case.
2. The code is accompanied by an automatic or manual test.
3. The code is written using the Python Enhancement Proposal (PEP) 8 style guide for Python code.^44^
4. The code documents its intended use case, input parameters, and expected output.

More specifically, each new feature and bug fix must add a unit test that tests the functionality of the feature. If automatic unit testing is not possible (e.g., because required third-party binaries are not available in the integrated testing environment), then a manual integration test is defined in which the feature is being used within a SIMPA simulation run. The output of the manual test is then reviewed by a SIMPA developer as a sanity check. Using such a mixture of automatic and manual tests, we aim to provide tests for every intended use case of SIMPA to ensure that the toolkit is stable and working as intended.

### 2.2 Software Architecture

**Fig. 2:**
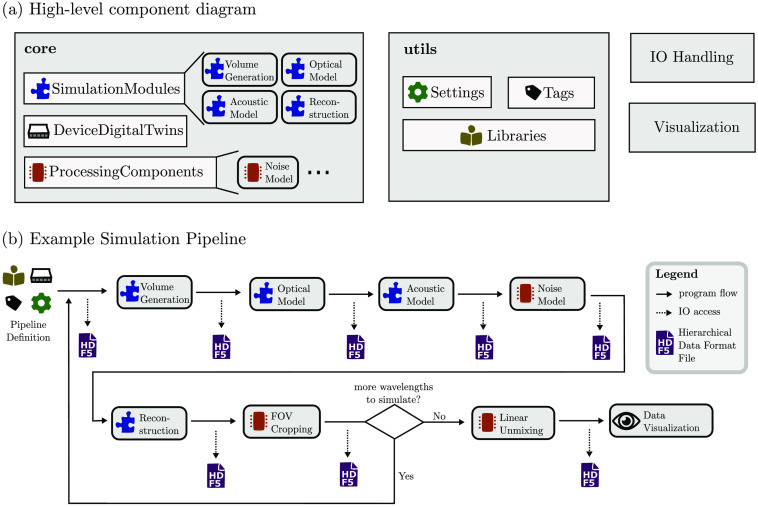
Software components of SIMPA. (a) The main software components of SIMPA’s software architecture. The toolkit consists of two main components, core and utils, as well as several smaller components (e.g., io_handling, visualisation), which are each composed of several subcomponents. The core contains all SimulationModules, DeviceDigitaltwins, and ProcessingComponents. The utils component contains the Settings dictionary, a standardized list of Tags, various Libraries, and other utility and helper classes to facilitate using the toolkit. (b) An example simulation pipeline. The pipeline is defined via a Settings dictionary using a standardized list of Tags. During the pipeline execution, each pipeline element (which can be either a SimulationModule or a ProcessingComponent) is called sequentially. After each step, the new results are amended to a hierarchical data format 5 (HDF5) file. The pipeline is repeated for each wavelength; afterwards, all multispectral ProcessingComponents are executed, and the results can be visualised. In this example, the included pipeline elements are volume generation, optical modeling, acoustic modeling, noise modeling, image reconstruction, field of view (FOV) cropping, linear unmixing, and result visualisation.

SIMPA provides a unified abstract data structure that combines existing simulation tools to represent the full signal generation process of a given optical and/or acoustic imaging modality. Specifically, SIMPA handles the data/information flow from and to each simulation tool and provides an infrastructure to use these tools in an integrated pipeline. Figure 2 shows the main components of SIMPA and visualises their interactions in an example simulation pipeline.

SIMPA contains two primary Python modules: core and utils. Furthermore, the toolkit features several smaller Python modules: io_handling, log, visualisation, algorithms, examples, and tests. The SIMPA core defines a centralized structure to provide simulation tool-specific adapters to the abstract modules for each step in the simulation process. The utils package provides a collection of libraries and convenience methods to help a researcher set up a customized simulation pipeline.

#### 2.2.1 Core

The core is organized into three Python submodules. The SimulationModules submodule provides interfaces for all simulation modules (e.g., the ones that are required for complete PA forward modeling). To meet the modularity criterion (Sec. 2), it contains abstract module definitions for the major parts: VolumeCreationModule, OpticalForwardModule, AcousticForwardModule, and ReconstructionForwardModule. Furthermore, the core contains a ProcessingComponents submodule that contains a base for components that supplement the main simulation modules, such as a component for noise modeling, which currently supports a number of noise models: salt and pepper noise, Gaussian noise, Poisson noise, uniform noise, and Gamma noise. Finally, the DeviceDigitalTwins submodule also contains base classes that enable the definition of digital twins of PA imaging devices such as slit or pencil illuminations combined with circular or linear detector geometries. Details on the digital device representation in SIMPA can be found in Sec. 2.3.

The main entry point for the user is the simulate method that is contained in the core. This method is responsible for the execution of all desired simulation modules and processing components (referred to as pipeline_elements).

To meet the extensibility criterion (Sec. 2), a developer has the freedom to add custom new simulation module adapters, processing components, or digital device twins. Each pipeline element in the simulation pipeline has to be fully self-contained and thus handle its produced result correctly within the information flow of SIMPA. To ensure this, each of the Python submodules provides an abstract class that encapsulates parts of the functionality. For example, a user can define a custom simulation module using the abstract SimulationModule class as a blueprint. To implement a Python adapter, it has to inherit from this class and overwrite the implementation method. Internally, the representation of the computational grid is defined by isotropic voxels. This does not necessarily exclude external tools that work on differently defined grids such as anisotropic voxels or mesh-based methods if the according adapter translates one into another. The edge size of the voxels is generally defined by the user attribute SPACING_MM, but this would not prevent an adapter from resampling the voxel sizes. The grid uses the default unit for length within SIMPA, which is mm. The default unit for time in SIMPA is ms.

#### 2.2.2 Utils

The utils Python module contains utility classes such as the Tags and Settings classes. The Settings class is a dictionary that contains key-value pairs defining the simulation parameters. To assert standardized naming conventions of the dictionary keys, these keys are globally accessible via the Tags class. Furthermore, there is the Libraries package that provides both LiteratureValues as well as collections of classes that represent, e.g., geometrical shapes and biochemical molecules. The LiteratureValues are used to instantiate these classes for the purpose of generating synthetic tissue models. The Libraries package provides the following collections:

LiteratureValues: Reference values for optical, acoustic, and morphological tissue properties including the respective online source.
SpectrumLibrary: Classes based on a Spectrum. A Spectrum represents wavelength-dependent tissue properties such as optical absorption or scattering defined for a specific set of wavelengths (depending on the reference literature).
MoleculeLibrary: Classes based on a Molecule. The Molecule class is used to represent the optical and acoustic properties of biochemical molecules such as melanin or hemoglobin.
TissueLibrary: Predefined MolecularComposition classes. A MolecularComposition is a linear mixture of different Molecules. The elements of the TissueLibrary are defined such that the optical and acoustic properties of the mixed Molecules agree with the literature references (e.g., skin or blood).
StructureLibrary: Classes based on a Structure. Each Structure defines the geometry of a certain volumetric shape (such as cuboids, tubes, or vessel trees) in a voxelized grid.

The interplay of these libraries is described in greater detail in Sec. 2.4. All libraries are designed such that they are easily customisable by the user, for example, by allowing for the addition of spectra, molecules, or tissue types.

#### 2.2.3 IO and data format

**Fig. 3:**
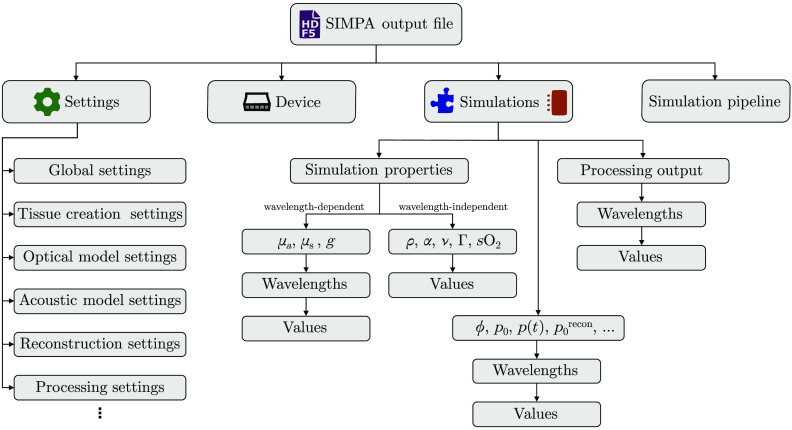
The SIMPA file data structure is hierarchical. The output file of SIMPA uses the Hierarchical Data Format 5 (HDF5). The top-level fields are (1) Settings in which the input parameters for the global simulation pipeline as well as for all pipeline elements are stored in. (2) The Device describes the digital device twin with which the simulations are performed. (3) The Simulations field stores all of the simulation property maps that serve as input for the pipeline elements, such as the optical absorption ($\mu_{a}$), scattering ($\mu_{a}$), and anisotropy (g). These properties are wavelength-dependent and therefore are saved for each wavelength respectively. The density (ρ), acoustic attenuation (α), speed of sound (ν), Grüneisen parameter (Γ) or blood oxygen saturation (${\mathrm{sO}}_{2}$) are wavelength-independent and therefore only stored once. The Simulations field also stores the outputs for each wavelength of each processing component and simulation module such as optical fluence (ϕ), initial pressure ($p_{0}$), time series pressure data (p(t)), or the reconstructed image ($p_{0}^{\mathrm{recon}}$). (4) The simulation pipeline is a list that stores the specific module adapters that have been combined and their order to form the simulation pipeline.

SIMPA uses the *Hierarchical Data Format 5* (HDF5)^45^ as it comprises a hierarchical data structure, has interfaces in many commonly used programming languages, and features the possibility of adding metadata. All inputs, settings, and outputs of a SIMPA simulation are stored in a central HDF5 file, and at the end of the simulation, the file contents can be repacked to be saved in a compressed manner. The SIMPA io_handling Python module abstracts from the communication with the h5py package^46^ and contains functionality to save and write data to the hard drive (Fig. 3).

SIMPA also offers the feature to export simulated time-series data into the data format proposed by IPASC.^47^ This data format is based on HDF5 as well and defines a standardized list of metadata parameters to include.^48^

### 2.3 Digital Device Twins

**Fig. 4:**
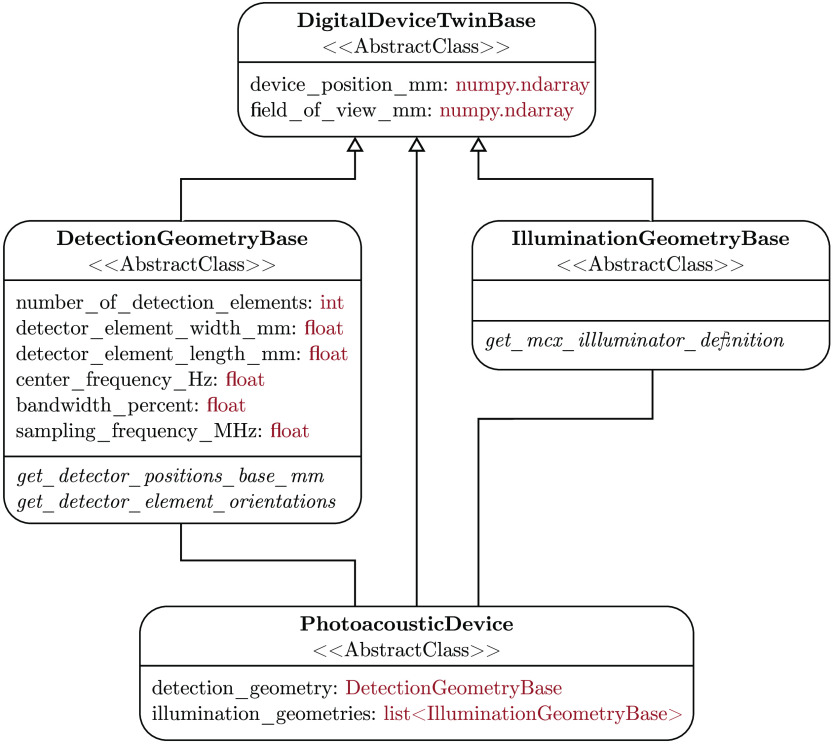
Unified modeling language (UML) class diagram of the digital device representation in SIMPA. Each box represents a class with the class name in bold. The first set of elements are the fields defined by these classes with their types shown in red, and the italic fields refer to abstract methods. A PA device comprises a detection geometry and an illumination geometry. All classes inherit from the DigitalDeviceTwinBase class, which defines common attributes: the device position and the FOV.

SIMPA enables the definition of digital twins of optical and acoustic devices by providing abstract base classes for the implementation of detectors and illuminators (cf. Fig. 4). To this end, SIMPA contains the DetectionGeometryBase and IlluminationGeometryBase classes, both of which inherit from the DigitalDeviceTwinBase class. The DigitalDeviceTwinBase class defines the device position and the field-of-view (FOV). The DetectionGeometryBase and IlluminationGeometryBase classes are responsible for defining the necessary parameters and abstract methods for the implementation of custom devices. To define a detection geometry or an illumination geometry, a class that inherits from the fitting base class and implements the necessary abstract methods needs to be written. A PA device is defined by having both a detection and an illumination geometry.

SIMPA predefines some commonly used detection and illumination geometries, as well as some PA devices. Currently, SIMPA provides classes for curved arrays, linear arrays, and planar array detection geometries, as well as disk, Gaussian beam, pencil beam, pencil array, and slit illumination geometries. Using these classes, the user can freely combine detection and illumination geometries as well as their relative positions to accurately represent real devices. SIMPA also provides digital twins of some PA devices: the multispectral optoacoustic tomography (MSOT) Acuity Echo, the MSOT InVision 256-TF, or the raster-scan optoacoustic mesoscopy (RSOM) Xplorer P50 from iThera Medical (iThera Medical GmbH, Munich, Germany). Because SIMPA currently only supports MCX as the optical forward model, and MCX only has a limited amount of supported illumination geometries, the MSOT Acuity Echo and the MSOT InVision 256-TF illumination geometries are represented by individual classes, and a version of MCX that supports these geometries is provided as a fork at: https://github.com/IMSY-DKFZ/mcx, (last visited March 22, 2022).

### 2.4 Diverse Tissue Modeling

**Fig. 5:**
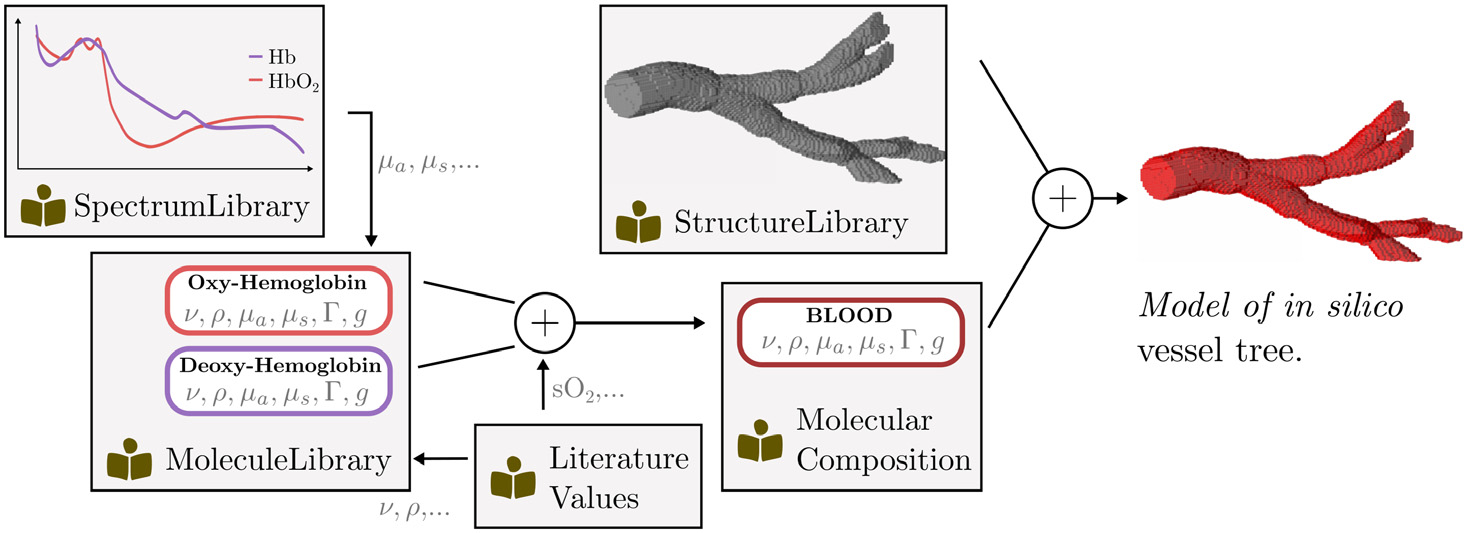
Overview of the steps involved for modeling an *in silico* vessel tree with SIMPA. The diagram shows the resources that SIMPA provides for users to create custom tissue models. Wavelength-dependent properties such as the optical absorption ($\mu_{a}$), scattering ($\mu_{s}$), or scattering anisotropy (g) are provided in the SpectrumLibrary, whereas wavelength-independent properties such as the speed of sound (ν), the tissue density (ρ), or the Grüneisen parameter (Γ) are provided by the LiteratureValues. A MolecularComposition corresponds to a linear mixture of Molecules that can be used in combination with a geometrical molecular distribution from the StructureLibrary to create an *in silico* model.

A core prerequisite for the simulation of realistic PA images is the modeling of diverse tissue geometries by generating plausible distributions of optical and acoustic parameters in a virtual volume. In this context, diversity comprises not only a wide variety of geometrical shapes that might occur in biological tissue but also the accurate modeling of optical and acoustic properties of different tissue types such as skin, blood, or fat. These tissue types are usually mixtures of molecules each with distinct properties, which can be difficult to represent computationally. To meet this need, SIMPA provides a VolumeCreation module that enables the convenient generation of custom tissue models. The backbone of the module is the way that the optical and acoustic tissue properties are represented using flexible MolecularCompositions (see Fig. 5). Using a hierarchical listing of predefined structures, the user can then create custom spatial distributions of these molecular compositions. The ability to create realistic tissue models depends on several factors, which include (1) the availability of high-quality reference measurements for the optical and acoustic properties, (2) information on the molecular composition of various tissue types, and (3) an accurate representation of their spatial distributions within the region of interest.

#### 2.4.1 Optical and acoustic molecule properties

A full list of all tissue properties considered in SIMPA is given in Tables 1–2. Within the SIMPA codebase, these molecular properties are integrated as inherent parts of a Molecule. While most properties can be approximated as singular values, the optical absorption, scattering, and anisotropy are wavelength-dependent and therefore represented by a Spectrum that linearly interpolates between the nearest known wavelengths to approximate the full spectrum during simulation.

**Table 1:** Overview of all optical properties that are represented in a SIMPA molecule with their respective units.

Optical properties	Unit
Absorption coefficient	${\mathrm{cm}}^{-1}$
Scattering coefficient	${\mathrm{cm}}^{-1}$
Scattering anisotropy	Unitless
Grüneisen parameter	Unitless

**Table 2:** Overview of all acoustic properties that are represented in a SIMPA molecule with their respective units.

Acoustic properties	Unit
Speed of sound	$m\;s^{-1}$
Density	$\mathrm{kg}\;m^{-3}$
Acoustic attenuation	$\mathrm{dB}\;{\mathrm{cm}}^{-1}\;{\mathrm{MHz}}^{-1}$

For wavelength-dependent information on the optical properties of the chromophores most commonly found in human tissue, Jacques published an invaluable review article^49^ and made information available via the OMLC website.^50^ We decided to follow the system of units introduced in the cited literature in SIMPA. For the tissue properties relevant for acoustic forward modeling, we used the IT’IS database for thermal and electromagnetic parameters of biological tissues^51^ as it provides information on the mean value and distribution of these properties for many different tissue types. Other literature sources that are being used by SIMPA for representing molecular properties are Kedenburg et al.^52^ for heavy water, Zhang et al.^53^ for water, or Antunes et al.^54^ for the optical properties of bone.

#### 2.4.2 Molecular tissue compositions

To represent the properties of a tissue type, SIMPA uses a MolecularComposition, which is a linear combination of Molecules. In the TissueLibrary, SIMPA provides several predefined tissue types such as blood, skin, muscle, and bone that are compiled from literature sources. However, the framework can also be used to easily define custom user-specific molecular compositions.

Information on the molecular composition of tissue types is sparse and scattered throughout the literature. SIMPA models the properties of different skin layers and muscle tissue using the review article of Bashkatov et al.,^55^ melanin content in the epidermis using Alaluf et al.,^56^ the water volume fractions of different tissue types in the human body using Timmins and Wall^57^ and Forbes et al.,^58^ and the distribution of arterial and venous blood oxygenations using Molnar and Nemeth^59^ and Merrick and Hayes.^60^

#### 2.4.3 Spatial tissue distribution

Taking the creation of an *in silico* forearm as an example, specialized clinical papers can be used to obtain information on aspects such as the distribution of sizes of the radial and ulnar artery^61^ and their accompanying veins,^62^ the thickness of skin layers such as the dermis and epidermis,^63^ the separation of the radius and ulna bones,^64^ and the depth of subcutaneous vessels.^65^

SIMPA offers the ability to create voxelized volumes of molecular compositions and provides two main ways to create their spatial distributions.

##### Model-based volume generator

The purpose of this Adapter is to enable a rule-based creation of voxelized simulation volumes. The generator is given a list of structures that are each represented by a voxelized definition of their shape, a molecular composition, and a priority. In the case of two structures occupying the same voxel, the molecular composition of the structure with the higher priority is chosen for that voxel. Based on their shape and priority, all structures are then merged into a single distribution of optical and acoustic parameters.

SIMPA provides a StructureLibrary that contains many basic 3D shapes (Structures), such as layers, spheres, elliptical tubes, cuboids, parallelepipeds, or vessel trees. These Structures can be mixed to create arbitrary simulation volumes. For the generation of vessel trees, we have integrated a random walk-based algorithm into SIMPA, in contrast to other work that uses Lindenmayer systems to build a grammar with the inclusion of stochastic rules.^66^

##### Segmentation-based volume generator

The purpose of this Adapter is to take voxelized segmentation masks as input and map them to specific tissue types, which allows for the easy inclusion of spatial tissue property distributions from other sources. The user themself is responsible for loading a segmentation mask from a file into memory and transforming it into a numpy array as an input for the SIMPA pipeline.

## 3 SIMPA Use Cases

The functionality spectrum covered by the SIMPA toolkit is best demonstrated by exemplary use cases. The use cases in this section build upon each other with increasing complexity. Section 3.1 introduces the initiation of a basic simulation pipeline. Section 3.2 shows the convenience of changing smaller hyperparameters of an existing pipeline and the impact on the outcome of the pipeline, and Sec. 3.3 analogously illustrates this for the change of whole simulation modules as well as digital device twins. Section 3.4 showcases the diversity of possible tissue geometries, and Sec. 3.5 compares simulation outcomes of SIMPA with a real PA image. Finally, Sec. 3.6 combines the previous sections to exemplify the generation of a diverse dataset of PA images. The optical and acoustic modeling toolkits used for all experiments in this section were MCX^24^ and k-Wave,^28^ using SIMPA-provided adapters. MCX uses the Monte Carlo method that repeatedly draws random variables from an underlying model distribution to reach high accuracy.^67^ MCX approximates a light transport model using this method. K-Wave is based on the k-space pseudospectral method for modeling nonlinear ultrasound propagation in heterogeneous media.^68^ All experiments were conducted using a workstation with an AMD(R) Ryzen 3900x 12-core central processing unit, 64 GB of RAM, and NVIDIA RTX 3090 GPU running Ubuntu 20.04., and they can be reproduced using the code available at https://github.com/IMSY-DKFZ/simpa_paper_experiments. The run times for each executable experiment are mentioned; however, a detailed analysis of SIMPA’s run times, computational requirements, and postprocessing examples^69^^,^^70^ can be found in the Supplementary Material.

### 3.1 Running a Simulation Out-of-the-Box

Simulations are run using the simulate function, which is located in the core Python module. The function simulate takes three input arguments: (1) a list with a definition of the simulation pipeline, (2) a Settings dictionary, which contains all parameters for the simulation, and (3) a Device, which represents a digital twin of a PAI device. The following listing shows how these three input parameters are defined and given to the simulate function. For each of the used simulation pipeline elements, a settings dictionary that contains the parameters needs to be defined. An overview of the user-side pseudocode to set up a simulation with SIMPA is given by:

import simpa as sp

# Create general settings

settings = sp.Settings(general_settings)

# Create specific settings for each pipeline element

# in the simulation pipeline

settings.set_volume_creation_settings(volume_creation_settings)

settings.set_optical_settings(optical_settings)

settings.set_acoustic_settings(acoustic_settings)

settings.set_reconstruction_settings(reconstruction_settings)

# Set the simulation pipeline

simulation_pipeline = [sp.VolumeCreatorModule(settings),

 sp.OpticalForwardModule(settings),

 sp.AcousticForwardModule(settings),

 sp.ReconstructionModule(settings)]

# Choose a PA device with device position in the volume

device = sp.CustomDevice()

# Simulate the pipeline

sp.simulate(simulation_pipeline, settings, device)

### 3.2 Customising Simulation Parameters

**Fig. 6:**
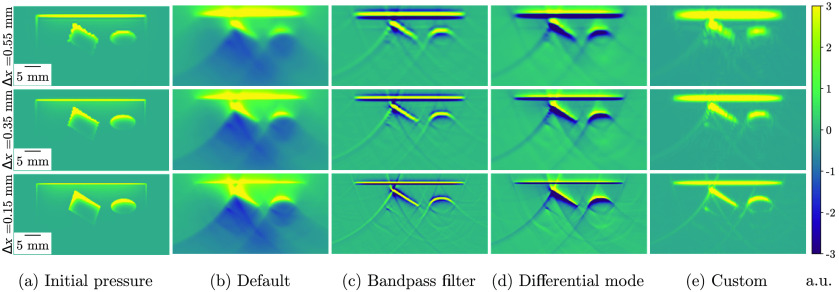
Simulation results with different hyperparameter configurations using a digital device twin of the MSOT Acuity Echo (iThera Medical GmbH, Munich, Germany). The results are shown for three spacings (Δx) in three rows (0.15, 0.35, and 0.55 mm), and from left to right, the columns show the following: (a) the ground truth initial pressure distribution; (b) the default pipeline with delay-and-sum reconstruction of the time-series pressure data (pressure mode); (c) delay-and-sum reconstruction with a bandpass filter (Tukey window with an alpha value of 0.5 and 1 kHz as high-pass and 8 MHz as low-pass frequencies) applied to the time-series data; (d) delay-and-sum reconstruction with the first derivative of the time-series data (differential mode); and (e) delay-and-sum reconstruction with a bandpass filter with the same configuration as in (c), the first derivative of the time-series data and envelope detection.

SIMPA enables easy customization of simulation parameters according to the criterion usability. A wide range of simulation outputs can be achieved by simply changing one parameter, such as the spacing or image reconstruction bandpass filter (Fig. 6), with the latter achieved, for example, by setting Tags.RECONSTRUCTION_PERFORM_BANDPASS_FILTERING in the reconstruction module settings to True instead of False as it is by default. To showcase this, a simulation pipeline was executed with three different spacings (0.15, 0.35, and 0.55 mm) and reconstructed with the default settings (delay-and-sum), with an applied bandpass filter, with a “differential mode” (delay-and-sum of the first derivative of the time signal), and finally, with a customized set of hyperparameters. For the bandpass filter, a Tukey window^71^ with an alpha value of 0.5 and 1 kHz as high-pass and 8 MHz as low-pass frequencies was applied. The set of hyperparameters was chosen such that the result is most similar to the underlying initial pressure. For different phantom designs, illumination geometries, or detection geometries, a different choice of parameters might be preferable.

The overall run time for these simulations was about 480 s. The run times of the optical and acoustic forward modules as well as the image reconstruction for the specified hardware are reported in Table 3. Only the mean for the different parameter combinations of these times are reported; however, an extensive listing of the run times of each module in each pipeline can be found in Tables S1-S4 in the Supplementary Material.

**Table 3:** Mean run times of the optical and acoustic forward modules and image reconstruction for simulation pipelines with different parameter combinations in seconds (s). The mean time was calculated from the run times of the pipelines: default, bandpass filter, differential mode, and custom. The times are reported for three different spacings: 0.15, 0.35, and 0.55 mm.

Spacing (mm)	Optical modeling time (s)	Acoustic modeling time (s)	Image reconstruction time (s)
0.55	1.19	7.96	2.22
0.35	2.80	8.28	2.21
0.15	27.77	11.62	2.22

### 3.3 Rapid Prototyping with Multiple Pipelines

**Fig. 7:**
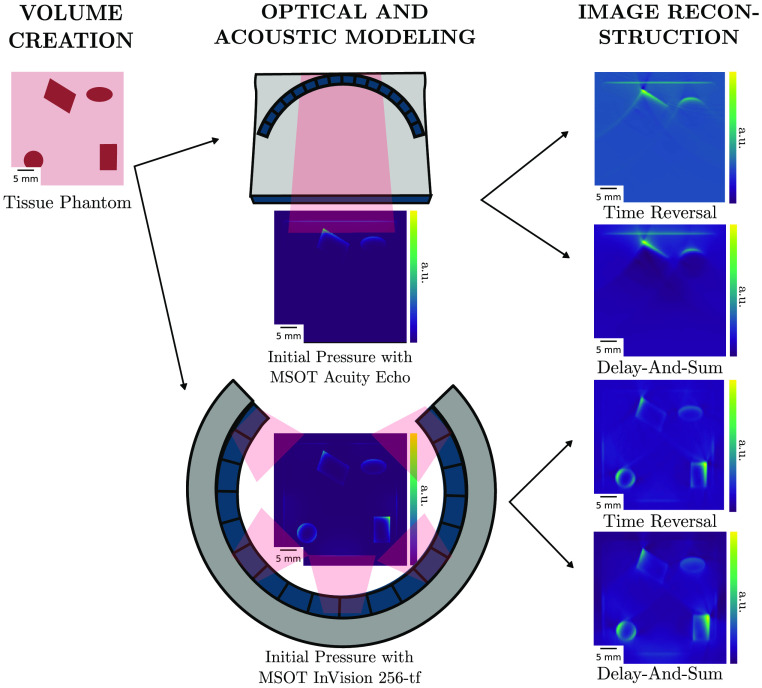
Demonstration of the versatility of the toolkit. From the same tissue phantom, two initial pressure distributions and time-series data are simulated using completely different PA digital device twin [in this case, the MSOT Acuity Echo and the MSOT InVision 256-TF (iThera Medical GmbH, Munich, Germany)]. The simulated time-series data are then reconstructed using different reconstruction algorithms (time reversal and delay-and-sum), resulting in four distinct simulation results.

SIMPA facilitates simulation of phantom imaging, which is highly relevant for experimental planning and rapid prototyping. To demonstrate this, a pipeline was executed with two commercial PAI systems (MSOT Acuity Echo and the MSOT InVision 256-TF devices, iThera Medical GmbH). For each device, the optical and acoustic forward simulations were executed only once. With the produced time-series data as a result, for each device, two different reconstruction adapters were added to the pipeline to reconstruct the final PA images. Currently, the following reconstruction algorithms are supported: delay-and-sum,^29^ delay-multiply-and-sum,^31^ signed delay-multiply-and-sum,^72^ and time reversal.^73^ The results that are shown in Fig. 7 are reconstructed with delay-and-sum or time reversal. The overall run time for these simulations was about 320 s with a spacing of 0.15 mm.

Not only does the user have the ability to easily exchange devices and module adapters in the simulation pipeline but the pipeline can also be designed in such a way that the simulation is executed efficiently. The optical and acoustic forward models had to be simulated only once for each device, and the two image reconstruction algorithms were applied afterward, demonstrating the modularity of SIMPA.

### 3.4 Generating Diverse Tissue Geometries

**Fig. 8:**
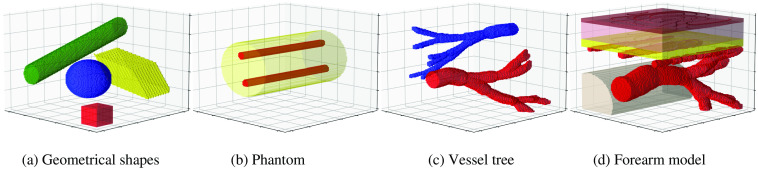
Examples of chromophore distributions that can be created using the SIMPA volume generation module. (a) Arbitrarily placed and oriented geometrical structures, i.e., a tube (green), a sphere (blue), a parallelepiped (yellow), and a cuboid (red); (b) a cylindrical phantom (yellow) with two tubular inclusions (red); (c) a vessel tree with high blood oxygen saturation (red) and a vessel tree with lower blood oxygen saturation (blue); and (d) a forearm model including the epidermis (brown), dermis (pink), fat (yellow), vessels (red), and a bone (gray).

A wide range of *in silico* tissue models can be generated using SIMPA. For this, we specifically showcase tissue structure distributions aligned to different use cases from the literature. Figure 8(a) shows an arrangement of different geometrical shapes such as cuboids and spheres as used by Cox et al.^69^ In Fig. 8(b), a cylindrical phantom with two absorbing inclusions, comparable to the one presented by Hacker et al.,^74^ is generated. A volume containing complex vessel trees can easily be generated similar to the human lung vessel dataset acquired from computed tomography used by Bench et al.^14^ as shown in Fig. 8(c). Lastly, realistic tissue models such as a human forearm used by Gröhl et al.^13^ are possible by combining the previously mentioned structures, which are shown in Fig. 8(d). The overall run time for these simulations was about 10 s.

### 3.5 Simulating Realistic Photoacoustic Images

**Fig. 9:**
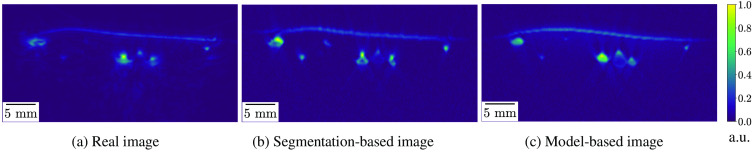
Comparison of simulations using SIMPA with a real PA image of a human forearm. From left to right, the panels show: (a) the normalized reconstructed PA image of a real human forearm acquired with the MSOT Acuity Echo; (b) a simulated image using SIMPA’s segmentation-based volume creator with a reference segmentation map of (a); and (c) a simulated image using SIMPA’s model-based volume creator. For both volume creators, a digital device twin of the MSOT Acuity Echo was used. For easier comparison, all images were normalized from 0 to 1 in arbitrary units.

Being as realistic as possible is key to many applications in which simulated data are needed. Nevertheless, it has been reported multiple times that a domain gap exists between simulated and real PA images.^3^^–^^5^^,^^14^ By its modular nature, SIMPA can be used to simulate PA images with a high degree of realism. To visually demonstrate the capabilities of the current version of SIMPA in this regard, an image of a human forearm was recorded from a volunteer using the MSOT Acuity Echo. The measurement was conducted within a healthy volunteer study that was approved by the ethics committee of the medical faculty of Heidelberg University under reference number S-451/2020, and the study is registered with the German Clinical Trials Register under reference number DRKS00023205. Based on this real image, the model-based, as well as the segmentation-based volume creators were used to synthetically recreate this image with SIMPA to compare the results with the original image (Fig. 9).

For the segmentation-based volume creator, the original image was manually annotated, and the different classes were assigned tissue properties by trial and error, so the image as a whole looks as close to the original image as possible. Using the model-based volume creator, the volume of the original image was recreated using the basic geometrical structures as described in Sec. 2.4. It should be mentioned that the model-based recreation of high-quality images, such as the one depicted in Fig. 9, is relatively time-consuming as it requires substantial manual interaction. To address this resource bottleneck and thus pave the way for the generation of large (training) data sets as required by modern machine learning algorithms, SIMPA also offers the option of generating the simulation volumes from predefined sets of rules (see Sec. 3.6). The results show that both of these methods can lead to images that closely resemble the real PA image. The overall run time for these simulations was about 80 s.

### 3.6 Generating a Diverse Dataset of Photoacoustic Images

**Fig. 10:**
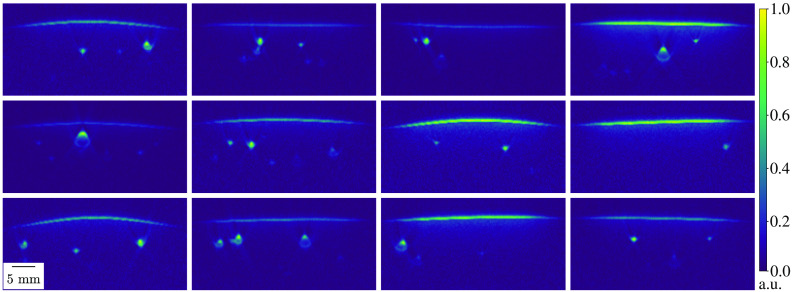
Example of a diverse dataset of simulated PA images. With randomized settings of amount, location, size, shape, and blood oxygen saturation of vessels as well as the curvature of the skin, 12 diverse PA images were generated and normalized between 0 and 1 in arbitrary units (a.u.). The spacing of all images was 0.15 mm. For all simulations, a digital device twin of the MSOT Acuity Echo was used.

For the training and the generalization ability of a complex deep learning model, a large and diverse dataset is crucial. In PAI, however, a vast amount of real PA images with ground truth annotations for their underlying properties such as optical absorption *in vivo* is not feasible. To remedy this, SIMPA can be used to generate an arbitrarily large dataset of simulated PA images with a degree of realism that can be seen in the previous section. In Fig. 10, 12 diverse PA images were simulated using randomized tissue mimicking settings of SIMPA’s model-based volume creator.

These randomized settings allow for controlled distributions of, for example, amount of vessels, vessel locations, skin curvature, and blood oxygen saturations. The overall run time for the generation of these 12 images was about 570 s with a spacing of 0.15 mm. An investigation of adverse programming effects in SIMPA when generating such a dataset can be found in the Supplementary Material.

## 4 Conclusion and Discussion

In this work, we present SIMPA, an open-source software library that allows for the simulation and image processing of optical and acoustic imaging modalities taking into account user-specific requirements common in the community. Core to the toolkit is its modular design, which allows for a flexible definition of simulation and processing pipelines. To this end, SIMPA defines abstract interfaces for the necessary forward modeling steps that allow for the integration of arbitrary third-party simulation tools in addition to modules already implemented in SIMPA. It already includes interfaces to toolkits that are commonly used in the field, such as MCX^24^ and k-Wave,^28^ is open-source, and is actively maintained and improved. Furthermore, a strong emphasis has been placed on tissue modeling as the basis for each simulation. SIMPA provides methods and functionalities to generate numerical tissue models that incorporate optical and acoustic tissue properties by means of a dynamic definition of molecular compositions. Using PAI as an example, we show the simulation results for several typical SIMPA use cases. By generating a diverse dataset of PA images, we demonstrate that SIMPA can create simulations with a high degree of flexibility suitable for, e.g., training of deep learning algorithms.

The images simulated with SIMPA look realistic (Fig. 9); however, because of the vast number of modeling assumptions both within SIMPA and within the used forward models, throughout all forward modeling steps, there remains a domain gap between simulated and experimental measurements. Steps toward increasing the realism of simulated images have already been taken by including various noise models and diverse tissue geometries such as the deformability of structures. This enables horizontal layers to more closely resemble the deformation of skin and vessels can thus also be squeezed analogously to applying pressure with an imaging device. Despite these efforts, computational modeling inaccuracies such as device-specific artifacts or a heterogeneous background with, e.g., varying blood volume fraction and oxygen saturation are not yet included.

SIMPA’s modular design also facilitates the exchangeability of simulation algorithms without affecting the integrity of the simulation pipeline. Because of the modular design, arbitrary pipeline elements can be added to the simulation. SIMPA provides example scripts to achieve this and comprises an extensive test suite that incorporates unit tests for the code, as well as manual test scripts that can be used to test the integration of forward models. Analysis of over 100 subsequent runs shows that sequential simulations do not affect each other; the detailed results can be found in the Supplementary Material.

Decreasing the potential for user error and lowering the barrier to entry for PA simulation is one of the core ideas behind SIMPA; hence, we show here the simulation and customization of specific use cases. SIMPA itself also contains many example scripts and documentation. The SIMPA developers try to ensure high code quality through its software development life-cycle, which includes the presence of tests, as well as internal code reviews before changes are integrated. Using SIMPA lowers the barrier of entry into the field of PA image simulation by taking over many of the researchers’ responsibilities in navigating the respective simulation tools. At the same time, this increased ease of use comes at the cost of a reduced amount of flexibility, as users are limited to the SIMPA interface and do not directly control the third-party tools. Despite the high level of abstraction, there is still room for user errors that can potentially be hard to identify. For support, researchers can open issues in the SIMPA GitHub repository and can also join the SIMPA Slack channel upon request.

Two major contributions of this work are the model-based volume creator that enables the user to create diverse spatial distributions of tissue properties and the segmentation-based volume creator that loads segmentation masks. The model-based approach includes features such as the simulation of partial volume effects and the rendering of the model in different spacings. Furthermore, it is straightforward to create diverse tissue geometries using random variables during the creation process (see Sec. 3.6). SIMPA provides many utility functions that make the model-based volume creator easy to use. Rendering the scene description into a voxelized grid, however, can become computationally expensive for small spacings, and the user is limited by the SIMPA-defined structure primitives (unless they want to implement their own Structure classes). The segmentation-based approach addresses this issue by featuring great flexibility in the shapes that it can simulate. Moreover, the creation of the voxelized grid is generally much faster. On the negative, the spacing of the simulation is limited to the spacing of the segmentation, which can lead to hard edges and staircase artifacts.

In addition to the signal simulation steps detailed in this paper, SIMPA also provides postprocessing modules for image processing. SIMPA currently provides two algorithms: (1) an iterative qPAI algorithm, implemented based on the publication of Cox et al. from 2006^69^ (cf. Fig. S3 in the Supplementary Material 4.1), and (2) a linear spectral unmixing algorithm based on singular value decomposition (cf. Fig. S4 in the Supplementary Material 4.2).

Future work will include supporting more forward models, such as numerical approximations of the radiative transfer equation for photon transport in biological tissue;^75^ supporting other optical imaging modalities such as multi-/hyperspectral diffuse reflectance imaging; the addition of more reconstruction algorithms; the capabilities for ultrasound simulation; and the provision of more digital commercial PA devices from a variety of vendors including distinct artifacts that are introduced by different devices. The IPASC is working on a standardiszed data format for PAI (Ref. 48) and has a digital device definition embedded in its format. They are currently planning to integrate support for their definition of the devices into MCX (available at: https://github.com/IPASC/PACFISH/issues/15, last visited March 22, 2022). Once this is achieved, we will support arbitrary illumination geometries within SIMPA. Furthermore, the variety of structures that can be used will be increased by including heterogeneous backgrounds that more closely represent the irregularities within tissue as well as larger, more complex, and connected structures that can represent organs or tumors. A great current challenge is the steep increase of needed computational resources, especially RAM and hard drive space, when decreasing the spacing of the computational grid. To this end, optimization strategies will be investigated to minimize the achievable spacing for a given hardware configuration. We only tested SIMPA with NVIDIA GPUs for GPU acceleration, but we plan to support a wider variety of computing platforms in the future. We are currently also working toward an interactive visualisation tool for the data and the addition of a graphical user interface for SIMPA, which could further flatten the learning curve. Other interesting avenues of future work could be the consideration of heterogeneous molecular distributions within the structures or the integration of state-of-the-art deep learning-based processing components or module adapters.

## Code, Data, and Materials Availability

The experiments conducted in this paper do not require any external data. The latest release of the SIMPA code can be downloaded from GitHub (https://github.com/IMSY-DKFZ/simpa, last visited 22 March 2022). The code used to generate the results and figures is available in a GitHub repository (https://github.com/IMSY-DKFZ/simpa_paper_experiments).
