# Supplementary material for: SIMPA: an open-source toolkit for simulation and image processing for photonics and acoustics
Source: J Biomed Opt. 2022 Apr 4;27(8):083010. doi: 10.1117/1.JBO.27.8.083010 (PMC8978263; doi:10.1117/1.JBO.27.8.083010)
Supplement: Supplementary file 1 [file JBO_027_083010_SD001.pdf]

Supplemental material for: SIMPA: An Open-source Toolkit for Simulation and Image Processing for Photonics and Acoustics

Janek Gröhl[1,†, now at 6,7], Kris K. Dreher[1,2,*,†], Melanie Schellenberg[1,3,4], Tom Rix[1,3], Niklas Holzwarth[1], Patricia Vieten[1,2], Leonardo Ayala[1,5], Sarah E. Bohndiek[6,7], Alexander Seitel[1,‡], and Lena Maier-Hein[1,3,5,‡]

[1]German Cancer Research Center, Computer Assisted Medical Interventions, Heidelberg, Germany
[2]Heidelberg University, Faculty of Physics and Astronomy, Heidelberg, Germany
[3]Heidelberg University, Faculty of Mathematics and Computer Science, Heidelberg, Germany
[4]HIDSS4Health - Helmholtz Information and Data Science School for Health, Heidelberg, Germany
[5]Heidelberg University, Medical Faculty, Heidelberg, Germany
[6]Cancer Research UK Cambridge Institute, University of Cambridge, Robinson Way, Cambridge, CB2 0RE, U.K.
[7]Department of Physics, University of Cambridge, JJ Thomson Avenue, Cambridge, CB3 0HE, U.K.

ABSTRACT

This document contains materials and plots supplementing the paper: SIMPA: An open-source toolkit for simulation and image processing for Photonics and Acoustics. The experiments conducted in this paper do not require any external data. The latest release of the SIMPA code can be downloaded from GitHub (`https://github.com/CAMI-DKFZ/simpa`, last visited 14th December 2021). The code used to generate the results and figures is available in a GitHub repository (`https://github.com/CAMI-DKFZ/simpa_paper_experiments`) and via zenodo (made available upon publication).

*Send correspondence to K.K.D. k.dreher@dkfz-heidelberg.de
†These authors contributed equally
‡Shared last authorship

1. SIMPA MEMORY AND TIME SCALING

One major use case of SIMPA is to simulate large training datasets for machine learning algorithms. As such, the computational costs for the SIMPA simulations is of special interest. All experiments in this section were conducted using a workstation with an *AMD(R) Ryzen 3900x 12-core* central processing unit (CPU), 64 GB of RAM, and *NVIDIA RTX 3090* GPU running Ubuntu 20.04.

Generally, a higher resolution in medical imaging is advantageous for a physician to be able to differentiate between small structures maybe even in the submillimeter regime. For simulation of PA images, a finer computational grid can simulate higher frequencies of the acoustic waves*. Unfortunately, with a higher resolution, the computational costs can be extreme, such that it is not possible even for advanced scientific computers to simulate PA images arbitrarily highly resolved. The resolution of a SIMPA simulation is given by the isotropic voxel size or *spacing*. A simulation pipeline with the same parameters as the default simulation in section 3.2 of the main paper has been executed ten times each with decreasing spacing from 0.4 mm to 0.1 mm and the RAM usage, as well as the run time, for each pipeline element has been recorded. These simulations indicate what the SIMPA user can expect of the scaling of *Random Access Memory* (RAM) usage, as well as the run time of an example simulation pipeline of SIMPA with decreasing spacing (Figure S1). It is important to note that the absolute numbers of the run times of the simulation runs can vary vastly from device to device, however, the scaling of both RAM usage and run time with decreasing spacing should be consistent.

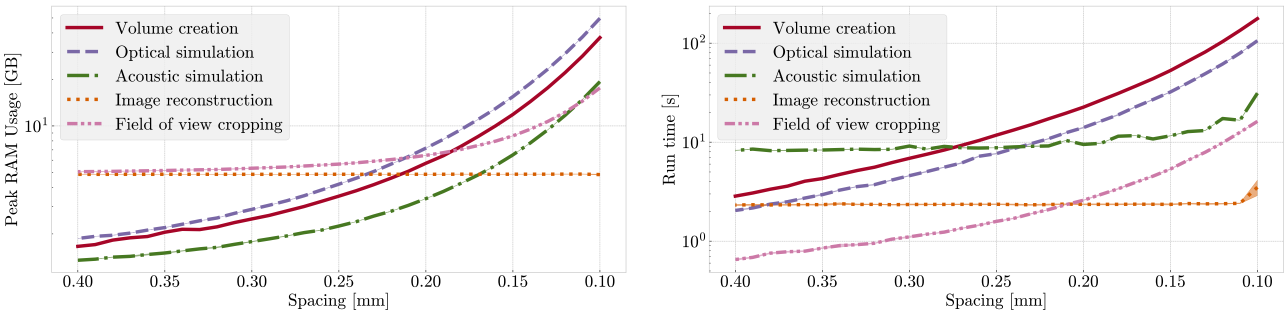

(a) Scaling of peak RAM usage with spacing.

(b) Scaling of simulation run time with spacing.

Figure S1: Analysis of the random access memory (RAM) and run time requirements of SIMPA. S1a shows the scaling of the peak RAM usage and S1b shows the scaling of the simulation run time with decreasing spacing from 0.4 mm to 0.1 mm. Both the peak RAM consumption in GigaBytes (GB) (S1a) and the run time scaling in seconds (s) (S1b) for each of the pipeline elements are shown on a logarithmic scale. The pipeline elements are colour-coded as follows: volume creation (red), optical modelling (purple), acoustic modelling (green), image reconstruction (orange), and field of view cropping (pink).

It should be noted that the **first** initialisation of any tensor on the GPU using PyTorch takes a lot longer (around two seconds) than the actual reconstruction algorithm. Because of this, the image reconstruction time including tensor initialisation is constantly around two seconds. As the actual reconstruction algorithm only takes about 20 ms, a reconstruction frame rate of 50 Hz can be achieved if the user initialises the PyTorch framework prior to executing the reconstruction algorithm.

*`http://www.k-wave.org/manual/k-wave_user_manual_1.1.pdf`, last visited 14th December 2021

2. CUSTOMISING SIMULATION PARAMETERS - RUN TIMES

In section 3.2 of the main paper, a simulation pipeline has been customised with various hyperparameters with the aim of reaching the most similar reconstruction result compared to the underlying initial pressure. In Tables S1, S2, S3, and S4 the run times for different parameter combinations can be found. The reconstructions in the different pipelines were performed with the default settings (delay-and-sum), with an applied bandpass filter, with a "differential mode" (delay-and-sum of the first derivative of the time signal), and finally, a customised set of hyperparameters. These times are reported for the spacings 0.15 mm, 0.35 mm, 0.55 mm.

Spacing [mm]	Optical modelling time [s]	Acoustic modelling time [s]	Image reconstruction time [s]
0.55	1.16	7.92	2.16
0.35	2.77	8.47	2.20
0.15	27.89	11.64	2.20

Table S1: Run times of the optical and acoustic forward modules and image reconstruction for the default simulation pipeline in seconds (s). The times are reported for three different spacings: 0.15 mm, 0.35 mm, 0.55 mm.

Spacing [mm]	Optical modelling time [s]	Acoustic modelling time [s]	Image reconstruction time [s]
0.55	1.17	7.91	2.28
0.35	2.77	8.15	2.20
0.15	28.05	11.70	2.25

Table S2: Run times of the optical and acoustic forward modules and image reconstruction in seconds (s) for a simulation pipeline with an applied bandpass filter (Tukey window with an alpha value of 0.5 and 1 kHz as high-pass and 8 MHz as low-pass frequencies). The times are reported for three different spacings: 0.15 mm, 0.35 mm, 0.55 mm.

Spacing [mm]	Optical modelling time [s]	Acoustic modelling time [s]	Image reconstruction time [s]
0.55	1.19	7.99	2.18
0.35	2.80	8.24	2.21
0.15	27.87	11.63	2.21

Table S3: Run times of the optical and acoustic forward modules and image reconstruction in seconds (s) for a simulation pipeline with delay-and-sum reconstruction with the first derivative of the time-series data . The times are reported for three different spacings: 0.15 mm, 0.35 mm, 0.55 mm.

Spacing [mm]	Optical modelling time [s]	Acoustic modelling time [s]	Image reconstruction time [s]
0.55	1.15	7.89	2.25
0.35	2.86	8.27	2.24
0.15	27.27	11.51	2.23

Table S4: Run times of the optical and acoustic forward modules and image reconstruction in seconds (s) for a simulation pipeline with delay-and-sum reconstruction with a bandpass filter (Tukey window with an alpha value of 0.5 and 1 kHz as high-pass and 8 MHz as low-pass frequencies), the first derivative of the time-series data and envelope detection. The times are reported for three different spacings: 0.15 mm, 0.35 mm, 0.55 mm.

3. INVESTIGATION OF ADVERSE PROGRAMMING EFFECTS

To simulate a large data set consisting of multiple photoacoustic images such as in section 3.6 of the main paper, the simulation of each image should run as fast as possible without influencing the simulation of a succeeding image of the dataset. This is relevant because the existence of dependencies within sequential runs would potentially lead to unusable or heavily biased data. The risk that this happens is high, as such dependencies could be introduced by minor software bugs (e.g. naming conventions are not kept, file access is not handled properly, instance variables are not reset properly, etc). It is therefore important to examine the behaviour of the toolkit in subsequent executions.

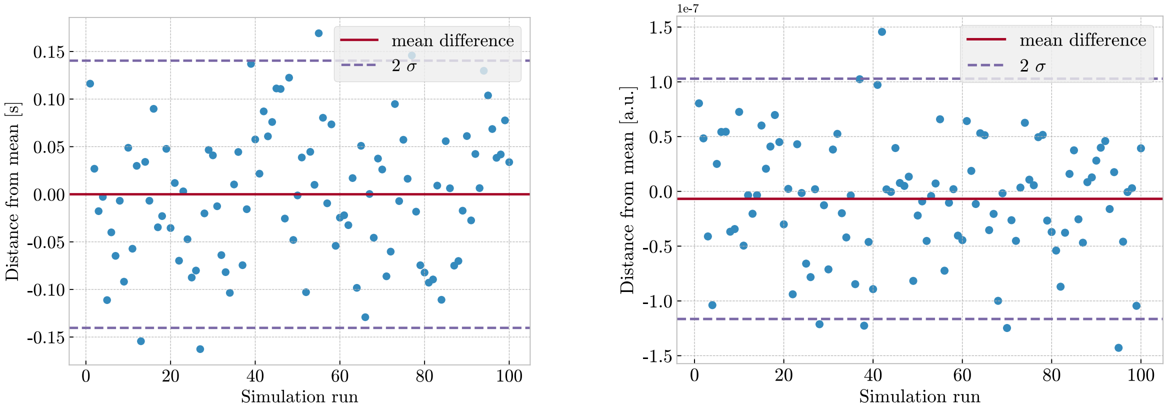

(a) Simulation run time differences. (b) Simulation result differences.

Figure S2: Analysis of the independence of subsequently executed simulations. Simulation run times of 100 sequential simulation executions are shown, where the pipeline was newly initialised every run. S2a shows the difference of the run times for each simulation in seconds (s). S2b shows the absolute differences between the simulation results, in this case the reconstructed images, where each image has been normalised between 0 and 1 in arbitrary units (a.u.). The dashed purple lines denote the $2\,\sigma$ interval around the mean and the red line denotes the mean difference.

To investigate a possible existence of adverse effects from subsequent simulations runs, 100 simulations with the same parameters are sequentially executed (with a spacing of 1 mm). The simulation run times are highly comparable (Figure S2); the simulation of the largest outlier took 0.17 s longer which is about 2% of the mean simulation time (≈ 7.95 s). As for the resulting simulated PA images, the difference between the mean image and largest outlier is about 1.5e-7 a.u. which is negligible since the images are normalized between 0 and 1 a.u.

4. PHOTOACOUSTIC IMAGE PROCESSING

Currently, there are two widely used image processing algorithms implemented in SIMPA and are demonstrated below.

4.1 Iterative quantitative photoacoustic imaging

The qPAI method introduced by Cox et al. 2006 uses a simple iterative scheme to recover optical absorption coefficients of a known initial pressure distribution by repeatedly simulating the optical fluence based on previous assumptions of the absorption coefficients and updating them accordingly until the algorithm converges. In comparison to the 2D method presented in the original paper, in SIMPA, a slightly advanced 3D method was implemented with MCX as the optical forward model. To reproduce the results obtained by Cox et al., one of the example volumes was recreated and the optical model was simulated. The initial pressure and the optical scattering were then used as input for the iterative qPAI algorithm. The ground truth and estimated absorption coefficient distributions, a difference image and the estimated fluence are shown in figure S3 where the same colour map has been chosen to make a comparison to the original paper easier. The results are surprisingly similar to the original paper even though we use a different optical forward model.

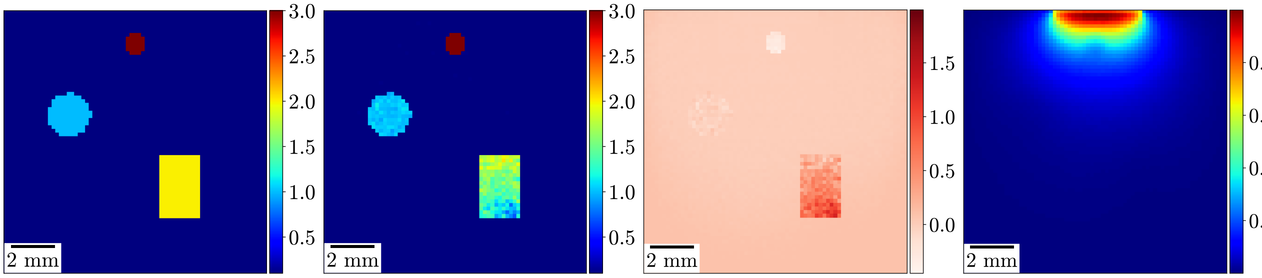

(a) Ground truth absorption [cm^{-1}]

(b) Recovered absorption [cm^{-1}]

(c) Difference of ground truth and recovered absorption [cm^{-1}]

(d) Recovered fluence [a.u.]

Figure S3: Recreation of the Cox et al. 2006 quantitative PA imaging experiment within SIMPA. From left to right, the panels show S3a the ground truth absorption coefficients in cm^{-1}; S3b the absorption coefficients recovered by the algorithm in cm^{-1}; S3c the absolute difference of S3a and S3b; and S3d the recovered light fluence of the last iteration step normalised between 0 and 1 in arbitrary units (a.u.). It has to be noted that while the original study was conducted using a 2D photon propagation model, a 3D model (MCX) was used in this case.

4.2 Linear unmixing

“Spectral unmixing is the decomposition of a mixed pixel into a collection of distinct spectra, or endmembers, and a set of fractional abundances that indicate the proportion of each endmember.” (Keshava et al., 2002) Linear spectral unmixing (the word *spectral* is often omitted) assumes that the pixel spectrum is a linear combination of the spectra of the endmembers. A version of linear spectral unmixing using singular value decomposition to reduce computational cost is implemented in SIMPA. To show the capabilities of this processing component, an example simulation was performed including linear unmixing using the wavelengths 750 nm, 800 nm, and 850 nm to provide an estimation of blood oxygen saturation (Figure S4). Notably, the errors of the estimated blood oxygen saturation are comparably low within the two ellipses especially in the upper area of the ellipses. The error is higher in the background because the signal of the highly absorbing structures dominates the spectrum of the reconstructed image.

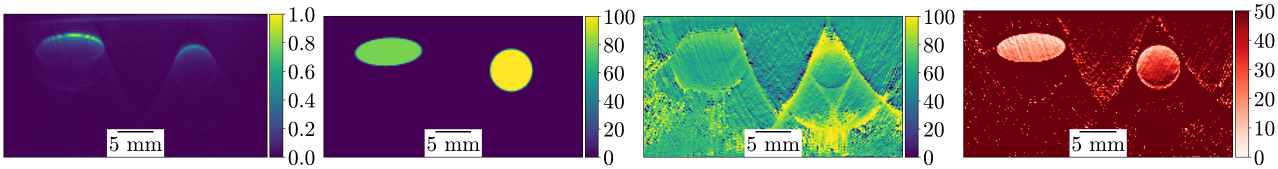

(a) Reconstructed image at 750 nm [a.u.] (b) Ground truth blood oxygen saturation at 750 nm [%] (c) Estimated blood oxygen saturation [%] (d) Absolute error [%]

Figure S4: An example application of linear unmixing on the reconstructed images within SIMPA. From left to right, the panels show: S4a the normalised reconstructed PA image at a wavelength of 750 nm which serves as input for the algorithm; S4b the ground truth blood oxygenation, where the background has 0%, the left structure 50%, and the right structure 100% oxygen saturation; S4c the estimated blood oxygenation after linear unmixing using 750 nm, 800 nm and 850 nm; and S4d the absolute error of the estimation when comparing S4b and S4c.
